# Conjoint and dissociated structural and functional abnormalities in first-episode drug-naive patients with major depressive disorder: a multimodal meta-analysis

**DOI:** 10.1038/s41598-017-08944-5

**Published:** 2017-09-04

**Authors:** Weina Wang, Youjin Zhao, Xinyu Hu, Xiaoqi Huang, Weihong Kuang, Su Lui, Graham J. Kemp, Qiyong Gong

**Affiliations:** 1 Huaxi MR Research Center (HMRRC), Department of Radiology, West China Hospital of Sichuan University, Chengdu, PR China; 2 Department of Psychiatry, West China Hospital of Sichuan University, Chengdu, Sichuan PR China; 3 Liverpool Magnetic Resonance Imaging Centre (LiMRIC) and Institute of Ageing and Chronic Disease, University of Liverpool, Liverpool, United Kingdom

## Abstract

Published MRI evidence of structural and resting-state functional brain abnormalities in MDD has been inconsistent. To eliminate interference by repeated disease episodes and antidepressant treatment, we conducted the first multimodal voxel-wise meta-analysis of studies of voxel-based morphometry (VBM) and the amplitude of low-frequency fluctuation (ALFF) in first-episode drug-naive MDD patients, using the Seed-based d Mapping method (SDM). Fifteen VBM data sets and 11 ALFF data sets were included. SDM-based multimodal meta-analysis was used to highlight brain regions with both structural and functional abnormalities. This identified conjoint structural and functional abnormalities in left lateral orbitofrontal cortex and right supplementary motor area, and also dissociated abnormalities of structure (decreased grey matter in right dorsolateral prefrontal cortex and right inferior temporal gyrus; increased grey matter in right insula, right putamen, left temporal pole, and bilateral thalamus) and function (increased brain activity in left supplementary motor area, left parahippocampal gyrus, and hippocampus; decreased brain activity in right lateral orbitofrontal cortex). This study reveals a complex pattern of conjoint and dissociated structural and functional abnormalities, supporting the involvement of basal ganglia-thalamocortical circuits, representing emotional, cognitive and psychomotor abnormalities, in the pathophysiology of early-stage MDD. Specifically, this study adds to Psychoradiology, an emerging subspecialty of radiology, which seems primed to play a major clinical role in guiding diagnostic and treatment planning decisions in patients with mental disorder.

## Introduction

Major depressive disorder (MDD) is predicted to be the leading cause of disability in high-income countries by the year 2030^1^. It is important to understand the early-stage abnormalities of MDD in the processing and regulation of emotions. Widely-accepted models suggest that MDD is underpinned by structural and functional abnormalities in multiple neuronal circuits, such as the fronto-limbic circuitry^2^ and the default mode network (DMN)^3^, and are generally supported by evidence from neuroimaging, notably magnetic resonance imaging (MRI).

There are several MRI analytic approaches to quantifying structural abnormalities, including traditional hand-drawn regions of interest (ROIs) and whole-brain morphometrics. The ROI method has substantial anatomical validity, but has two major limitations: it is time-consuming and vulnerable to ROI selection bias^4^. There are two whole-brain analytic methods for quantifying structural abnormalities: voxel-based morphometry (VBM) and vertex-based morphometry. Vertex-based morphometry is often applied to cortex thickness. In the present work we wished to focus on GM volume, for which VBM is well-suited. VBM is an automated whole-brain technique which calculates local concentrations of GM in an unbiased way without *a priori* specification of ROIs^5^. GM volume reduction has been reported in anterior cingulate cortex (ACC)^6^, orbitofrontal cortex (OFC)^7, 8^, and dorsolateral prefrontal cortex (DLPFC)^6, 9^, which are all prefrontal regions involved in the automatic regulation of emotional behavior^10^. Structural alterations have also been reported in hippocampus^11, 12^ and amygdala^9^, the key regions of the limbic system theoretically identified as important in mood regulation in the pathophysiology of MDD. Additionally, a GM abnormality in the cerebellum^13^, which is involved in cognitive processing, has been reported in MDD^14^.

MRI methods for identifying functional abnormalities are broadly of two kinds: resting-state functional MRI and task-based functional MRI. Task-based functional MRI maps specific brain regions recruited during a target-detection task designed to evaluate responses to target stimuli^15^. However, task-related changes in neural activation represent only a small fraction of the brain’s total activity^16, 17^, because intrinsic activation is energetically more costly than responses to external stimuli^18^. Knowing how the brain allocates the majority of its resources is therefore essential for understanding neural mechanisms associated with MDD. In addition, there is a lack of agreement about task paradigms^19^. Resting-state MRI analytic methods to define the local features of the spontaneous BOLD signal include amplitude of low-frequency fluctuation (ALFF) and regional homogeneity (ReHo): ALFF quantifies the intensity of low-frequency oscillations in spontaneous neural activity^20, 21^ while ReHo reflects the statistical similarity of spontaneous neural activity among spatially adjacent brain tissues^22^. Because of its physiological correlates^23^, ALFF is a more direct index of regional spontaneous neuronal activity, and can be used to locate specific impaired brain regions^24, 25^. ALFF also helps to avoid the potential bias induced by selection of the ‘seed’ voxels or the number of components in resting-state functional connectivity analysis^24, 26^ such as graph theory, ROI-to-ROI matrix analysis, seed-to-voxel analysis and independent component analysis. Resting-state studies in MDD have reported increased ALFF in the frontal cortex, including ACC, OFC^27, 28^ and posterior cingulate cortex/precuneus^29^, as well as the fusiform gyrus^29–31^ and lingual gyrus^30, 32^, which have been thought to reflect the excessive self-referential processing of MDD. Decreased ALFF has been reported in the cerebellar hemispheres^33, 34^ and superior temporal gyrus^32, 35^, and this has been linked to deficits in cognitive control of emotional processing. Reduced ALFF in the OFC has also been reported in MDD^31, 36^.

To date, volumetric and resting-state functional differences have been inconsistent and are poorly replicated for some brain regions. This is partly explained by considerable variation between studies in sample size (limiting the power to detect subtle brain differences and yielding both false-positive and false-negative findings^37^), in patients’ demographic and clinical characteristics, and in imaging protocols. We aimed to conduct a whole-brain voxel-wise meta–analysis to explore in a preliminary way the most robust findings across a range of published VBM and ALFF studies. Furthermore, to report on multimodally affected brain regions (the frontal-limbic regions where both structural and functional alterations have been reported), we performed an additional meta-analysis to display abnormalities in both VBM and ALFF in a single map. There are, we suggest, two ways to look at this analysis. On the working assumption that both modalities reflect a common pathophysiology, it is important to know that putatively-affected brain regions show conjoint abnormalities of both GM and brain function in MDD. The most plausible interpretation is that functional abnormalities observed in MDD are mediated by the underlying structural abnormalities^38^, potentially by complementary mechanisms where the direction of structural and functional changes are opposite. Alternatively, clearly dissociated abnormalities may throw an interesting light on pathophysiology. One such study applied VBM and ALFF together in drug-naive MDD, finding decreased GM in the parietal-temporal regions and decreased ALFF in the temporal regions and cerebellum^39^; however, no overlap was observed in the same template^39^. One reason for such failure to detect conjoint GM and brain function abnormalities might be that, hypothetically, structural damage in one region might cause functional abnormality in another. Alternatively, it might be simply an artifact of the relatively small sample size. In either case, a multimodal meta-analysis approach to identifying conjoint abnormalities from VBM studies of GM volume and ALFF studies of brain activity in MDD should be illuminating.

Other factors may contribute to the variability among MRI results in MDD. Antidepressant medication might increase heterogeneity and limit the interpretability and generalizability of the results, especially in the light of evidence that drugs may have important effects, such as upregulating neurotrophin expression^40^, altering neuronal remodeling^41^ and protecting against GM loss^42, 43^, in both animal and human studies^44–46^. In addition, studies on the course of the illness have reported brain structure and function differences between patients with first-episode (FE) and recurrent depression. For instance, compared with patients with recurrent MDD and with healthy controls, patients with FE depression showed increased amygdala volume^47^. However, depressed subjects with multiple depressive episodes showed hippocampal volume reductions which were not found in FE patients^48^. In view of this we restricted our analysis to FE and drug-naive MDD patients to eliminate interference by repeated episodes and antidepressant treatment.

In the present meta-analysis, we provide an up-to-date quantitative summary of studies investigating GM and ALFF abnormalities in FE drug-naive MDD patients, using Seed-based d Mapping (formerly “Signed Differential Mapping”) (SDM)^49^, a new version of effect-size signed differential mapping (ES-SDM)^50^ which has previously been applied to e.g. studies of dementia with Lewy bodies^51^, childhood maltreatment^52^, alcohol dependence^53^, and migraine^54^. Furthermore, we used a multimodal meta-analytical method integrated into SDM which enables combination of the results of the separate meta-analyses conducted from studies using different modalities to detect brain regions which display both structural and functional abnormalities^55^; this has previously been applied to studies of subjects at familial high risk for schizophrenia^56^, obsessive-compulsive disorder^57^ and FE psychosis^50^, but this seems to be its first application in MDD.

In brief, we conducted separate meta-analyses of VBM studies and ALFF studies on FE drug-naive MDD, followed by a multimodal meta-analysis of VBM studies and ALFF studies on FE drug-naive MDD to determine whether individuals exhibit brain regions with both structural and functional abnormalities.

## Results

### Included studies and sample characteristics

**Figure 1:**
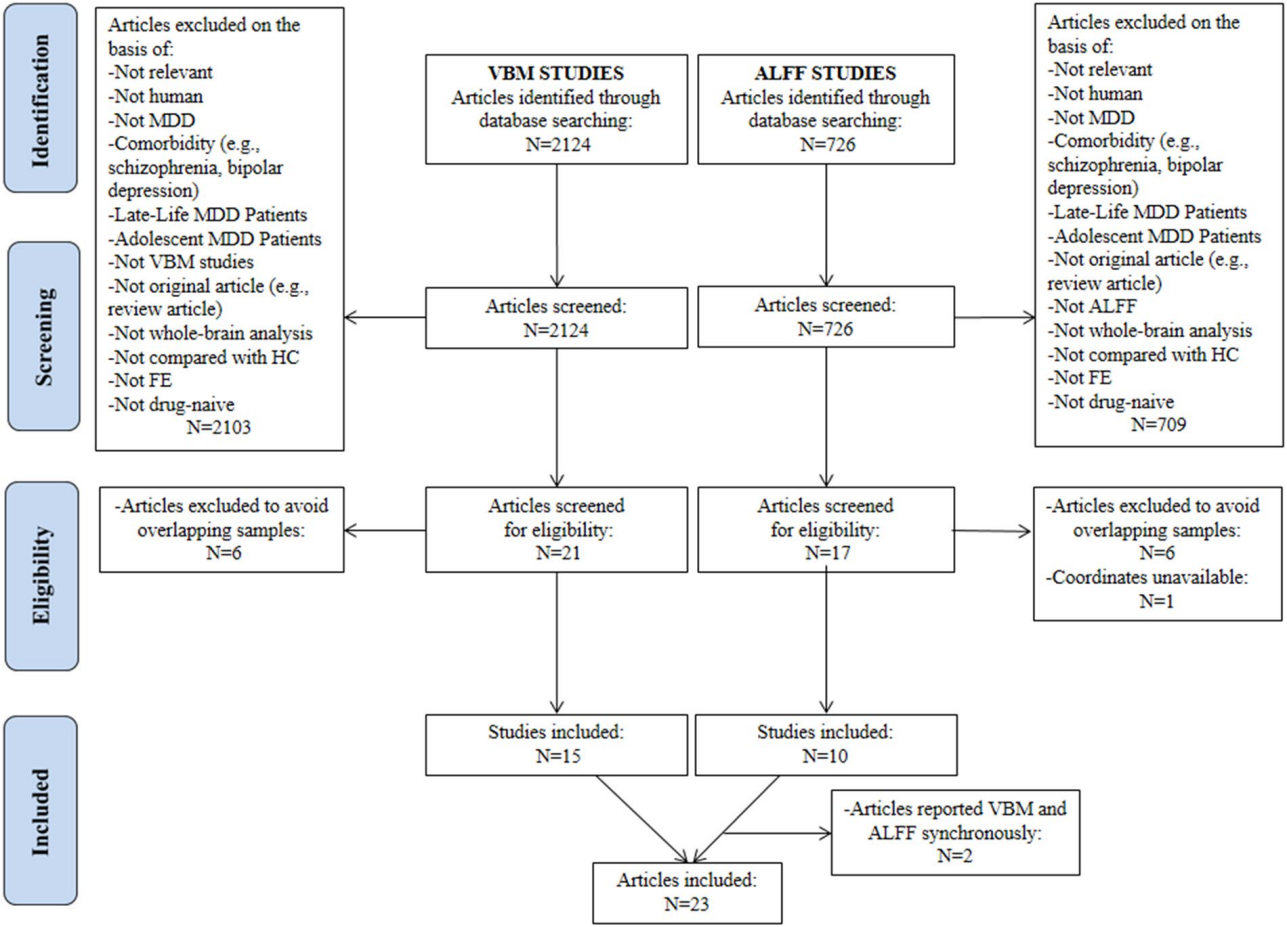
Meta-analysis of voxel-based morphometry and amplitude of low-frequency fluctuation studies in first-episode drug-naive patients with major depressive disorder. Study selection was done according to “Preferred reporting items for systematic reviews and meta-analysis” (PRISMA) guidelines. *Abbreviations*: ALFF, amplitude of low-frequency fluctuation; FE, first-episode; HC, healthy control; N, number; MDD, major depressive disorder; VBM, voxel-based morphometry.

Figure 1 shows a flow diagram of the identification and attrition of studies. The search strategy identified 2124 structural and 786 functional neuroimaging studies. Though we did not apply any language restriction in the search, all the abstracts yielded were in English; any articles in other languages were translated into English or Chinese for assessment. Of the 38 studies which met our inclusion criteria, we excluded 12 which reanalyzed previously published data, 1 in which coordinates were unavailable, and 2 which reported VBM and ALFF results synchronously, leaving 23 peer-reviewed and published original studies^10, 11, 19, 30, 33–36, 39, 58–71^. One of the ALFF studies analysed two different subgroups of MDD patients, namely early treatment responsive and nonresponsive patients^33^, both compared with the same HC group; each subgroup comparison was included as a data set in the present meta-analysis. Specifically, the structural meta-analysis included 15 data sets of 15 VBM studies with 471 FE drug-naive MDD subjects (194/277 male/female; mean age 34.0 years), matched with 521 controls (226/295 male/female; mean age 33.6 years); the resting state functional imaging meta-analysis included 11 data sets of 10 ALFF studies with a total of 261 FE drug-naive MDD subjects (126/135 male/female; mean age 31.9 years), matched with 278 controls (138/140 male/female; mean age 30.7 years). Table 1 and Table 2 summarise clinical and demographic data and technique details from all the included studies. The quality scores ranged from 9 to 12 (mean score 11.1), showing that these studies were of high quality. In no study was there any significant difference in age and sex between the MDD group and the HC.

**Table 1:** Demographic and clinical characteristics of subjects in the 23 voxel-based morphometry data sets included in the meta-analysis.

Study	Number (female)		Age (y)		Illness Duration	Severity	Mood State	Mean Episodes	Drug Status	Quality Scores
	MDD	HC	MDD	HC	(years)	(scale type)				(out of 12)
**VBM**										
Cheng *et al*., 2010	68(47)	68(47)	30	31	0.92	22 (HDRS)	depressed	FE	drug-naive	11.5
Guo *et al*., 2014	24(11)	44(24)	26	24	0.41	26 (HDRS)	depressed	FE	drug-naive	11
Kong *et al*., 2014	28(17)	28(14)	34	32	0.18	22 (HDRS)	depressed	FE	drug-naive	12
Lai *et al*., 2015	53(28)	54(29)	40	40	0.42	22 (HDRS)	depressed	FE	drug-naive	12
Liu *et al*., 2011	15(15)	30(30)	43	41	NA	37 (HDRS)	depressed	FE	drug-naive	10
Liu *et al*., 2012	17(7)	17(7)	27	24	0.22	26 (HDRS)	depressed	FE	drug-naive	10.5
Lu *et al*., 2016	30(15)	26(13)	34	31	NA	24(HDRS)	depressed	FE	drug-naive	11.5
Qiu *et al*., 2014	46(33)	46(33)	35	35	0.33	23 (HDRS)	depressed	FE	drug-naive	10
Ide *et al*., 2015	38(21)	42(18)	48	43	NA	21 (HDRS)	depressed	FE	drug-naive	11.5
Tang *et al*., 2007	14(14)	13(13)	30	30	0.45	≥18(HDRS)	depressed	FE	drug-naive	10.5
Tang *et al*., 2011	35(18)	35(18)	28	29	NA	28 (HDRS)	depressed	FE	drug-naive	11.5
Wang et al., 2012	18(9)	18(9)	34	35	0.42	25 (HDRS)	depressed	FE	drug-naive	11.5
Watanabe et al., 2015	29(13)	45(12)	45	41	NA	21 (HDRS)	depressed	FE	drug-naive	11.5
Zhang *et al*., 2012	33(16)	32(15)	21	21	NA	38 (CES-D)	depressed	FE	drug-naive	11.5
Zou *et al*., 2010	23(13)	23(13)	31	37	0.65	24 (HDRS)	depressed	FE	drug-naive	12
**ALFF**										
Du *et al*., 2016	16(11)	18(8)	39	35	NA	49(CTQ)	depressed	FE	drug-naive	10
Guo *et al*., 2012	17(7)	17(7)	27	24	0.22	26 (HDRS)	depressed	FE	drug-naive	11.5
Guo *et al*., 2014	24(11)	24(10)	26	24	0.41	26 (HDRS)	depressed	FE	drug-naive	11
*Wang *et al*., 2014-END	30(13)	33(14)	36	32	0.46	25 (HDRS)	depressed	FE	drug-naive	11
#Wang *et al*., 2014-ERD	26(10)	33(14)	33	32	0.33	28 (HDRS)	depressed	FE	drug-naive	11
Wang *et al*., 2012	18(9)	18(9)	34	35	0.42	25 (HDRS)	depressed	FE	drug-naive	11.5
Xu *et al*., 2010	14(6)	14(6)	29	30	NA	≥17(HDRS)	depressed	FE	drug-naive	9
Yan *et al*., 2014	14(14)	18(18)	36	33	0.33	26 (HDRS)	depressed	FE	drug-naive	11
Zhao *et al*., 2014	51(27)	50(28)	28	29	NA	≥17(HDRS)	depressed	FE	drug-naive	11.5
Zhang *et al*., 2014	32(18)	35(17)	21	21	NA	38 (CES-D)	depressed	FE	drug-naive	11.5
Zhu *et al*., 2012	19(9)	18(9)	56	54	0.29	24 (HDRS)	depressed	FE	drug-naive	10

*Abbreviations*: ALFF, amplitude of low-frequency fluctuation; CES-D, Center for Epidemiological Studies depression scale; END, treatment-nonresponsive; ERD, treatment-responsive; FE, first-episode; HC, healthy control; HDRS, Hamilton depression rating scale; MDD, major depressive disorder; NA, not available; VBM, voxel-based morphometry.

**Table 2:** Technique details of VBM and ALFF studies on MDD in meta-analysis.

Study	MRI scanner	Software	Smoothing(FWHM)	p-value	voxels	Coordinates
**VBM**						
Cheng *et al*., 2010	1.5 T	SPM5	8mm	p < 0.001(uncorrected)	50	1
Guo *et al*., 2014	3.0 T	SPM8	8mm	p < 0.001(GRF)	NA	1
Kong *et al*., 2014	1.5 T	SPM8	8mm	p < 0.05(FDR)	50	4
Lai *et al*., 2015	3.0 T	FSLVBM	7.5mm	p < 0.05(FEW)	40	6
Liu *et al*., 2011	3.0 T	SPM8	6mm	p < 0.05(FEW)	100	1
Liu*et al*., 2012	1.5 T	SPM8	8mm	p < 0.001(uncorrected)/p < 0.05(FEW)	50	15
Lu *et al*., 2016	3.0 T	FSLVBM	7.5mm	p < 0.05(FEW)	NA	0
Qiu *et al*., 2014	3.0 T	SPM8	8mm	p < 0.05(FDR)	50	6
Ide *et al*., 2015	3.0 T	SPM8	8mm	p < 0.05(FDR)	NA	0
Tang *et al*., 2007	1.5 T	SPM5	8mm	p < 0.05(MCC)	25	2
Tang *et al*., 2011	3.0 T	SPM5	NA	p < 0.05(NA)	100	5
Wang *et al*., 2012	3.0 T	SPM5	4mm	p < 0.05(MCS)	NA	3
Watanabe *et al*., 2015	3.0 T	SPM8	8mm	p < 0.05(FDR)	NA	11
Zhang *et al*., 2012	1.5 T	SPM8	8mm	p < 0.05(FEW)	15	2
Zou *et al*., 2010	3.0 T	SPM2	NA	p < 0.05(MCC)	NA	2
**ALFF**						
Du et al., 2016	3.0 T	SPM	6mm	p < 0.05(ASC)	17	4
Guo *et al*., 2012	1.5 T	SPM8	8mm	p < 0.05(FDR)	10	5
Guo *et al*., 2014	3.0 T	SPM8	8mm	p < 0.001(GRF)	20	2
Wang *et al*., 2014	3.0 T	SPM8	8mm	p < 0.001(uncorrected)/p < 0.05(ASC)	15	9
Wang *et al*., 2012	3.0 T	SPM5	4mm	p < 0.05(MCC)	40	6
Xu *et al*., 2010	3.0 T	SPM2	4mm	p < 0.005 (uncorrected)	20	4
Yan*et al*., 2014	3.0 T	DPARSF2	4mm	p < 0.05(ASC)	18	3
Zhao *et al*., 2014	3.0 T	SPM5	8mm	p < 0.001(uncorrected)	10	2
Zhang *et al*., 2014	1.5 T	SPM8	8mm	p < 0.05(FEW)	50	4
Zhu *et al*., 2012	1.5 T	SPM5	8mm	p < 0.05(MCS)	46	8

*Abbreviations*: ALFF, amplitude of low-frequency fluctuation; ASC, AlphaSime correction; FDR, false discovery rate; FWE, family-wise error correction; GRF, Gaussian random field; HC, healthy control; HDRS, Hamilton depression rating scale; MCS, Monte Carlo simulations; MCC, multiple comparison correction; MDD, major depressive disorder; NA, not available; VBM, voxel-based morphometry.

### Changes in regional grey matter

**Figure 2:**
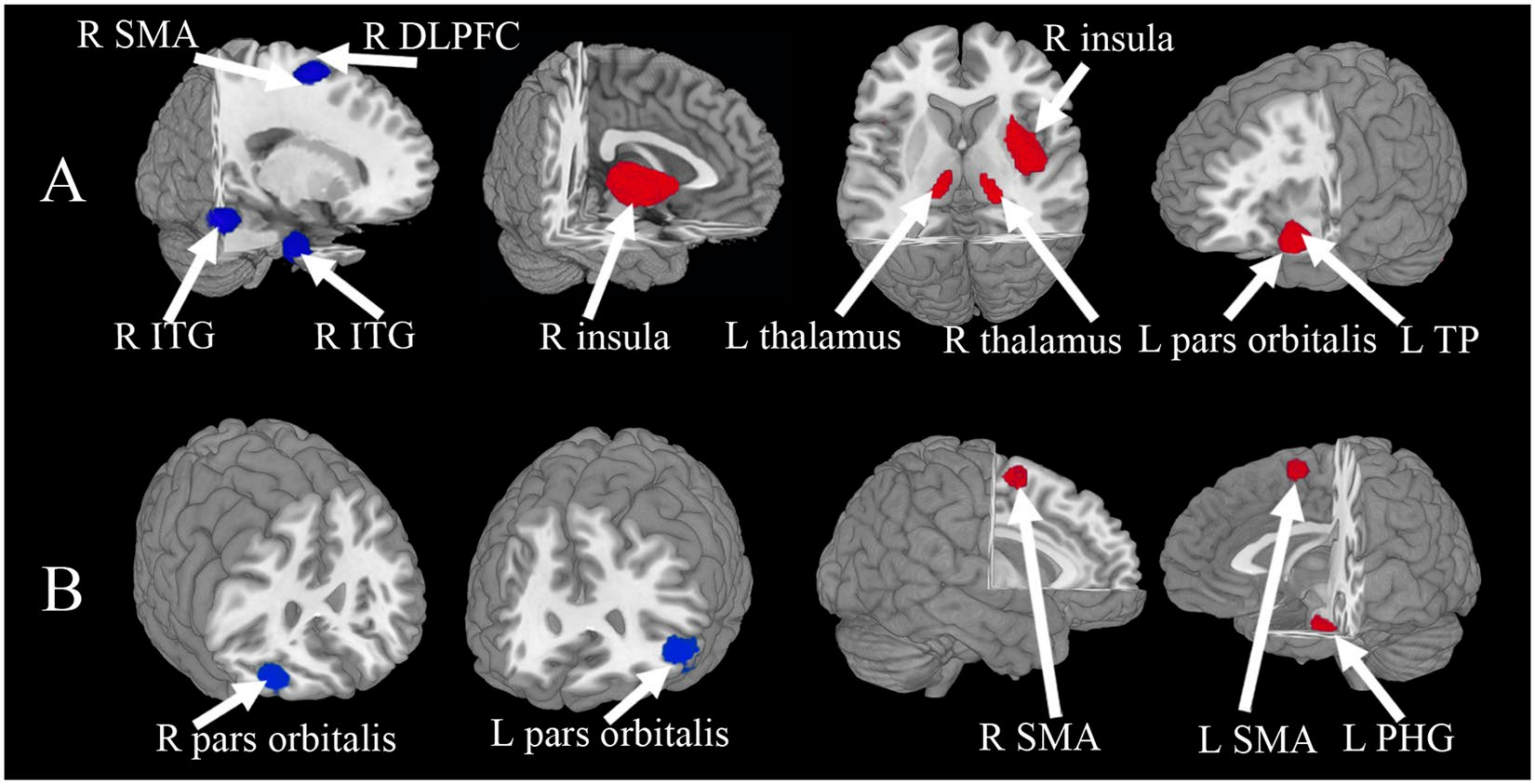
Areas of increased (red) and decreased (blue) grey matter or resting-state brain activity in first-episode drug-naive with major depressive disorder compared with healthy controls in the meta-analyses of (**A**) voxel-based morphometry studies and (**B**) amplitude of low-frequency fluctuation studies. *Abbreviations*: DLPFC, dorsal lateral prefrontal cortex; ITG, inferior temporal gyrus; L, left; PHG, parahippocampal gyrus; R, right; SMA, supplementary motor area; TP, temporal pole.

A group comparison of FE drug-naive MDD patients with HC across the 15 data sets in the main meta-analysis of VBM studies revealed decreased GM relative to controls in right DLPFC, right supplementary motor area (SMA), and right inferior temporal gyrus (ITG) extending to fusiform gyrus, and increased GM relative to controls in the right insula extending to putamen and striatum, left lateral OFC, left temporal pole (TP), and bilateral thalamus (Table 3 and Fig. 2).

**Table 3:** Regions of significant differences in GM and brain activity between patients with major depressive disorder and healthy controls.

Region	Maximum				Cluster breakdowns	Jackknife
	MNI Coordinates x, y, z	SDM z-score	P value uncorrected	Number of voxels		
**GM**						
*Decreased GM*						
R superior frontal gyrus, dorsolateral, BA 6	18, 0, 62	−1.340	0.000046432	184	R superior frontal gyrus, dorsolateral, BA 6 (184)	14/15 (Cheng *et al*.)
R supplementary motor area, BA 6	8, 2, 64	−1.468	0.000045248	65	R supplementary motor area, BA 6 (65)	14/15 (Cheng *et al*.)
R inferior temporal gyrus, BA 20	44, −12, −34	−1.061	0.000577986	249	R inferior temporal gyrus, BA 20 (155). R fusiform gyrus, BA 20 (94)	14/15 (Liu *et al*., 2012)
R inferior temporal gyrus, BA 37	62,−50, −16	−1.045	0.000619292	216	R inferior temporal gyrus, BA 20 (119)R inferior temporal gyrus, BA 37 (94)R middle temporal gyrus, BA 20 (3)	14/15 (Liu *et al*., 2012)
*Increased GM*						
R insula, BA 48	36, −4, 10	1.462	0.001414061	603	R insula (283)R lenticular nucleus, putamen, BA48 (225)R striatum (60)R rolandic operculum, BA 48 (35)	13/15 (Kong *et al*.; Tang et al.,2011)
L inferior frontal gyrus, orbital part, BA 47	−46, 20, −10	1.465	0.001460493	76	L temporal pole, superior temporal gyrus, BA 38 (44)	13/15 (Watanabe *et al*.; Tang et al.,2011)
L temporal pole, superior temporal gyrus, BA 38	−52, 18, −12	1.501	0.001088917	178	L temporal pole, superior temporal gyrus, BA 38 (152)L insula, BA 48 (26)	13/15(Watanabe *et al*.;Tang *et al*.,2011)
L thalamus	−12, −18, 10	1.509	0.001083791	38	L thalamus (38)	10/15(Cheng *et al*.; Kong et al.; Guo *et al*.; Ide *et al*.; Qiu *et al*.)
R thalamus	16, −24, 10	1.559	0.000789583	47	L thalamus (44)R pons (6)R hippocampus (3)	11/15(Guo *et al*.; Ide *et al*.; Qiu *et al*.; Zhang et al.)
**Brain activity**						
*Decreased brain activity*						
L inferior frontal gyrus, orbital part, BA 47	−52, 34, −10	−1.138	0.001785636	361	L inferior frontal gyrus, orbital part, BA 47 (233)L inferior frontal gyrus, triangular part, BA 45 (100)L middle frontal gyrus, orbital part, BA 46 (28)	10/11 (Zhang *et al*.)
R middle frontal gyrus, orbital part, BA 11	32, 40, −16	−1.197	0.00178045	236	R middle frontal gyrus, orbital part, BA 47 (146)R middle frontal gyrus, orbital part, BA 11 (39)R inferior frontal gyrus, orbital part, BA 47 (51)	10/11 (Zhang *et al*.)
*Increased brain activity*						
L supplementary motor area, BA 6	−2, −2, 62	1.506	0.002317190	50	L supplementary motor area, BA 6 (30)L supplementary motor area (20)	10/11 (Zhang *et al*.)
R supplementary motor area, BA 6	4, 6, 60	1.473	0.002802312	63	R supplementary motor area, BA 6 (57)R supplementary motor area (6)	10/11 (Zhao *et al*.)
L parahippocampal gyrus, BA 36	−24, −12, −30	1.430	0.003659010	38	L parahippocampal gyrus, BA 36 (32)L hippocampus, BA 36 (6)	9/11 (Xu*et al*.; Du *et al*.)
**Multimodal analysis**						
*Increased GM but decreased brain activity*						
L inferior frontal gyrus, orbital part, BA 47	−46, 24, −8	2.127	~0	155		
*Decreased GM but increased brain activity*						
R supplementary motor	8, 2, 62	2.906	~0	267		
Area, BA 6						

*Abbreviations:* BA, Brodmann area; GM, grey matter; L, left; MNI, Montreal Neurological Institute Space; R, right; SDM, Seed-based d Mapping.

In whole-brain jackknife sensitivity analysis the findings of decreased GM in MDD patients in the right DLPFC, right SMA, and right ITG remained significant in all but 1 combination, and increased GM in the right insula, left lateral OFC, and left STG in all but 2 combinations. The right thalamus and left thalamus remained significant in all but 4 and 5 combinations, respectively (Table 3).

In analysis of heterogeneity the right insula and left TP with increased GM showed significant statistical heterogeneity between studies (p < 0.005), while the remaining regions with altered GM did not show significant between-study heterogeneity (p > 0.005) (see Supplementary Table S2).

In analysis of publication bias, the Egger test was significant in the right SMA (P = 0.034) and right ITG (P = 0.025) but not for right DLPFC (P = 0.051), right ITG (P = 0.112), right insula (P = 0.371), left lateral OFC (P = 0.645), left TP (P = 0.641), left thalamus (P = 0.971) or right thalamus (P = 0.936) in the VBM metaanalysis.

### Changes in resting state regional brain activity

The main meta-analysis of the ALFF studies on MDD patients showed significantly enhanced brain activities in the bilateral SMA and left PHG extending to hippocampus and attenuated brain activities in the bilateral lateral OFC (Table 3 and Fig. 2).

In whole-brain jackknife sensitivity analysis the findings of attenuated brain activity in the bilateral lateral OFC and SMA were highly replicable, being preserved throughout all but 1 combinations of the data sets. The results in left PGH remained significant in all but 2 combinations (Table 3).

In analysis of heterogeneity, the regions with altered brain activities did not showed significant statistical heterogeneity between studies (p > 0.005).

In analysis of publication bias, the Egger test was significant for left SMA (P = 0.031), right SMA (P = 0.027) and right PGH (P = 0.031), but not for the left lateral OFC (P = 0.870) and right lateral OFC (P = 0.928) with decreased brain activities in the brain activity meta-analysis.

### Multimodal analysis of grey matter and brain activity

**Figure 3:**
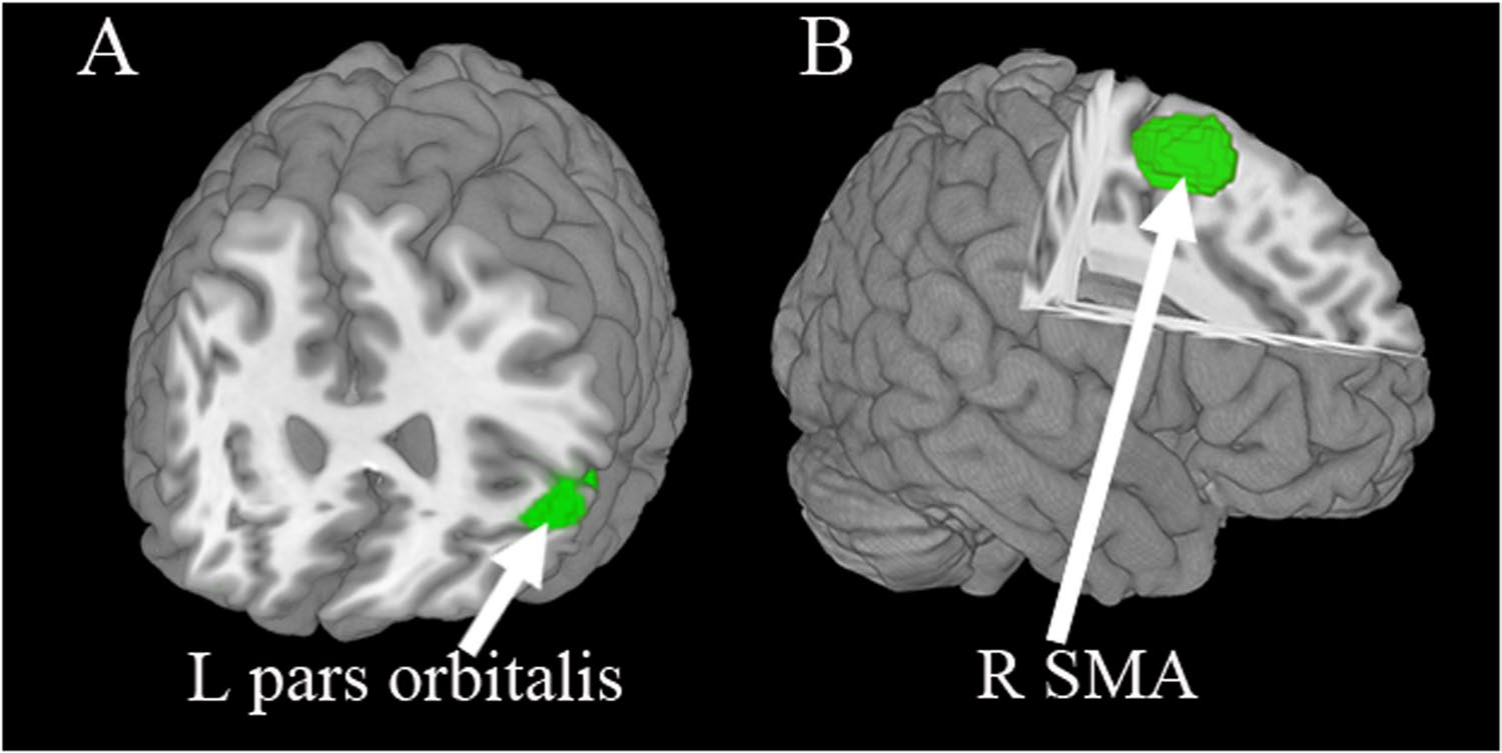
Multimodal meta-analysis reveals (**A**) increased grey matter with decreased brain activity in the left OFC and (**B**) Decreased GM with increased brain activity in the right SMA in first-episode drug-naive patients with MDD compared with healthy controls. *Abbreviations:* L, left; MDD, major depressive disorder; R, right; SMA, supplementary motor area.

The results were then summarised by putting structural and functional findings in a single meta-analytic map to demonstrate regions which showed both structural and functional abnormalities. This revealed increased GM with decreased brain activity in the left lateral OFC, and decreased GM with increased brain activity in the right SMA (Table 3 and Fig. 3). The left lateral OFC finding was observed in the two separate structural and functional meta-analyses and preserved in the jackknife sensitive analyses, and was without publication bias or heterogeneity. The right SMA finding was observed in the two separate structural and functional meta-analyses and preserved in the jackknife sensitive analyses, and was without heterogeneity, but failed in both publication bias analyses.

### Subgroup meta-analyses

Details of the results of subgroup analysis are presented in Supplementary Materials.

## Discussion

### Aims and strengths of the study

This is to our knowledge the first multimodal neuroimaging meta-analysis which attempts to localise the neural substrates of MDD by combining information from whole-brain VBM studies investigating GM with ALFF studies of spontaneous brain activity.

Methodological strengths are the novel techniques combining features from coordinate meta-analytic approaches and standard meta-analytic methods, and the multimodal approach. The restriction to FE MDD patients helps distinguish the intrinsic brain features of the disease from potential effects of episode times, and the restriction to drug-naive MDD patients minimises the interference from medication effects.

Both anatomical and functional brain abnormalities in MDD were observed, characterised by decreased GM mainly localizing in the right DLPFC, right SMA, and right ITG extending to the fusiform gyrus, and increased GM in the right insula extending to putamen and striatum, left lateral OFC, left TP, and bilateral thalamus, along with increased brain activity in the bilateral SMA and left PHG extending to hippocampus, and decreased brain activity in the bilateral lateral OFC. Because of the publication bias in the right SMA and right ITG findings of the VBM study, as well as in the bilateral SMA and right PHG findings of the ALFF study, these results should be interpreted with some caution. The multimodal meta-analysis identified conjoint structural and functional differences in the left lateral OFC and right SMA in MDD.

These main findings can be thought of in terms of three circuits (DLPFC-striatum-thalamus circuit, lateral OFC-striatum-thalamus circuit, and SMA-striatum-thalamus circuit) broadly representing emotion, cognition and motor dysregulation respectively^70^.

### Grey matter abnormalities in MDD

The most prominent finding was decreased GM in the right DLPFC, part of the central executive network which plays an important role in working memory and attention^72^, and is related to impaired cognitive function in FE drug-naive patients with MDD. This GM loss may reflect the reductions in glial cell density and neuronal size in the prefrontal cortex reported in postmortem studies^73, 74^. As the lateral prefrontal lobe is a well-known neural substrate involved in the pathophysiology of MDD, being associated with cognitive dysfunction^75, 76^, the GM loss in this region may be causally important. Furthermore, repetitive transcranial magnetic stimulation of the DLPFC is an established treatment for depression^77^. In rat models of depression, a lower expression of synaptic-function-related genes and correspondingly reduced number of synapses in the DLPFC has been reported^78^, and a similar phenomenon might underlie the decreased DLPFC volume in MDD.

The striatum has been associated with mood, cognitive processes and movement regulation^79^ and has connections with the DLPFC, OFC, SAM and temporal lobe^70^. The GM deficit in the putamen may contribute causally to the symptoms of MDD^80, 81^.

The thalamus is a complex structure, associated with the experience and expression of emotion in mood disorders^12^. We found symmetrical increased GM in thalamus in FE drug-naive MDD patients, consistent with a postmortem report of elevated neuron number in thalamus^82^, and also with a previous study in which increased GM in thalamus was related to pre-apoptotic osmotic changes or hypertrophy in FE drug-naive MDD patients^68^. However, structural studies and a previous meta-analysis have reported decreased GM in the thalamus^83, 84^. A possible explanation for this discrepancy may be that the latter enrolled data sets with a different course of illness or number of episodes. Consequently, we speculate that the increased volume of bilateral thalamus may be involved in the early stage of MDD, and is not likely to be the result of medication exposure.

MDD patients also showed increased GM in the right insula and left TP. The insula has extensive connections to several areas of the cortex and limbic system implicated in monitoring interceptive awareness^85, 86^, high-level cognitive control and attentional processes^87^. Increased insular activation to facial expressions of disgust in MDD may reflect an emotion processing bias^88^. Contrary to our finding, several studies reported reduced GM in insula^25, 89, 90^, and so this may be an effect of recurrent episodes^91^. Furthermore, increased GM in insula could be interpreted as resulting from neuroinflammation^92^: there is significantly elevated translocator protein density, an important aspect of neuroinflammation, in insula during a major depressive episode^92^. The TP is a visual and auditory-related brain region implicated in the processing of working memory and facial emotions^93^. Our findings are in line with previous reports of morphological alteration in the TP, an apparently early sign of MDD unlikely to result from treatment with antidepressants^94, 95^. Moreover, longitudinal MRI studies have reported progressive GM loss in the temporal lobe in MDD patients^95, 96^. Accordingly, the increased GM in right insula, bilateral thalamus and left temporal lobe might represent a specific character of early-stage MDD.

### Regional brain activity abnormalities in MDD

The meta-analysis of ALFF studies found increased spontaneous brain activity in the left PHG extending to hippocampus. Both structures belong to the limbic system and play a central role in regulation of emotions, motivation, memory, affective dimension of pain^6, 21, 23^ and cognitive processes in MDD^10^. Surprisingly, no differences in GM volumes were detected in the hippocampus despite the fact that a variety of studies have reported abnormalities in that region^12, 83^. Possible reasons for this are differences in illness duration, medication status, age of onset and the number of episode. The volume deficit of hippocampus reportedly correlates with illness duration^9, 84^, and decreased hippocampus volume was detected in patients with long illness duration compared with short duration^60^. A newly-published meta-analysis suggests that the lower hippocampus volume is associated with the number of episodes, whilst no difference was detected between FE MDD patients and controls^97^. In our meta-analysis, all patients were FE and drug-naive, and most of studies were of short duration, which may explain why we found no structural hippocampus abnormality.

### Subgroup meta-analyses

In addition to the results in the pooled meta-analysis, we found increased ALFF in the left posterior cingulate gyrus and right precuneus in subgroup meta-analyses of studies with large sample size. These regions belong to the DMN, which has a role in the balance between processing of external stimuli and internal and self-directed processing, which has long been thought to be involved in the pathophysiology of MDD. However, they failed in the pooled ALFF meta-analysis. This suggests that the detection of changes in DMN may be influenced by sample size; larger studies are needed to confirm this finding.

### Conjoint abnormalities in grey matter and brain activity in MDD

The multimodal meta-analysis identified conjoint structural and functional differences in the left pars orbitalis (increased GM with decreased brain activity), which is a part of the inferior frontal gyrus (Brodmann area 47), belonging to lateral OFC. The OFC, being important parts of the affective network, are involved in the emotional processing of mental states^98, 99^. However, there is a difference between the medial part and lateral part, processing negative and positive emotion separately^71^. This is reconcilable with the conceptualization of MDD as a disorder of emotion regulation^100, 101^. However, a number of studies have found decreased GM in this region^7, 102^, corroborated by a previous meta-analysis of volumetric MRI studies^79^. It should be noted that most previous studies included patients on antidepressant treatment, while we included only studies of drug-naive patients. We hypothesise that increased GM may be related to temporal hypertrophy^103^, marking areas of early neuronal pathology without the confounding factors of repeat episodes and treatment. One study has reported volume being larger at illness onset, and then declining with multiple episodes or treatment in mood disorder^103^. Regarding brain activity, the presence of anxiety symptoms of MDD is reportedly associated with decreased OFC activation^104^. In MDD the severity of depression correlates negatively with activity in the left lateral OFC^105^. Reduced baseline resting state connectivity within the orbitofrontal component was predictive of clinical response in medication-free MDD patients^106, 107^. Thus, the imbalance between structure and brain activity may represent a distinctive alteration of FE drug-naive MDD patients.

In addition, conjoint structural and functional differences were found in the right SMA (decreased GM with increased brain activity), which forms part of the SMA-striatum-thalamus circuit. This is traditionally considered the cortical area necessary for voluntary movement as well as implicated in psychomotor retardation^81^, the key feature of MDD, but it also participates in cognitive activities such as working memory^108^, implicit learning ability^109^, and attention and executive function^110^. As most of these are impaired in MDD, it is tempting to infer a causal link. Our finding was in accord with previous studies relating the reduced regional volumes of the right SMA to psychomotor retardation in early-onset depression patients^60, 109^. If a primary decrease of GM volume in SMA were accompanied by a compensatory hyperfunctionality of the remaining GM, involving higher regional cerebral metabolism^111^ and cerebral blood flow^112^, this would likely increase local ALFF. Conversely, primary hyperfunction might lead to a decrease in GM by glutamate-induced ‘excitotoxicity’^96^. Of particular importance, a previous review indicated that the reduced GM volume in some structures may produce partial volume effects in functional images^19^. For instance, MDD subjects relative to controls show metabolic activity that appears reduced in the subgenual prefrontal cortex^113^. However, when this anatomical deficit is taken into account by correcting the metabolic data for the partial volume averaging effect associated with the corresponding GM reduction, metabolism instead appears increased in the subgenual prefrontal cortex in the unmedicated-depressed patients^114^. Whatever the pathophysiology, the regions identified by the multimodal meta-analysis could serve as a specific ROIs template for both individual postmortem histopathological and *in vivo* imaging studies.

### Limitations

Firstly, combining numerous potentially underpowered studies with SDM meta-analysis using peak voxels, as we have done, may not reveal subtle widespread changes which are undetected by individual studies. A traditional meta-analysis, or an SDM meta-analysis using raw SPM images, gains much of its advantage by pooling raw data, including nonsignificant results, from all studies to increase power^115^. Our SDM meta-analysis using only significant co-ordinates as input is actually a tool for spatial integration of already-significant results. It is possible, given the hypothesis of subtle GM or brain activity abnormalities, that some areas of altered GM volume or brain activity do not reach significance in smaller studies but would prove significant if raw SPM maps were combined for SDM analysis.

Secondly, we discussed the findings that were not significant in the multimodal analysis as dissociated abnormalities in grey matter and brain activity in MDD. This dissociated distribution may not represent the real pattern of MDD abnormalities, because it may rather be due to failure to reach statistical significance in the multimodal analysis.

In the Egger test, we found publication bias in right SMA and right ITG in the VBM analysis, and in bilateral SMA and right PGH in the ALFF analysis, so another important limitation is the possibility of selective positive reporting and publication bias.

Finally, most of the primary studies have been so far conducted in China, thus limiting the generalizability of the current findings to other populations.

## Summary

The present meta-analysis revealed a complex pattern of neural abnormalities in first-episode drug-naive MDD patients, characterised by conjoint and dissociated structural and functional brain abnormalities in brain regions involved in motor, cognition and emotional processing. These volumetric and functional alterations support the notion that multiple parallel basal ganglia-thalamocortical circuits^70^, together with other limbic regions (parahippocampus, hippocampus) contribute to the underlying pathophysiology of early-stage MDD. First-episode drug naive MDD patients showed increase in GM as well as decrease in brain activity in the left lateral OFC, and decrease in GM as well as increase in brain activity in right SMA, which could therefore serve as a specific ROI template for future studies. Of note, this study adds to Psychoradiology (https://radiopaedia.org/articles/psychoradiology), an emerging subspecialty of radiology, which seems primed to play a major clinical role in guiding diagnostic and treatment planning decisions in patients with mental disorder^116, 117^.

## Methods

### Study selection

Meta-analysis was conducted according to the Preferred Reporting Items for Systematic reviews and Meta-Analyses guidelines (PRISMA)^118^. A systematic strategy was used to search for relevant studies published in PubMed, Embase, Web of Science, Science Direct and Google Scholar. Candidate structural imaging studies were sought using the keywords “depression” or “depressive” or “unipolar depression” or “major depression” or “major depressive disorder” or “MDD” plus “voxel-based morphometry” or “VBM” or “voxel*” or “morphometry”. Resting state functional imaging studies were sought using the keywords “depression” or “depressive” or “unipolar depression” or “major depression” or “major depressive disorder” or “MDD” plus “amplitude of low-frequency fluctuation” or “ALFF” or “low-frequency fluctuation” or “LFF”. The search was conducted up to July 2016, with no time-span specified for date of publication. Language of publication was not a specific search criterion. The reference lists of these studies were checked to identify further studies for inclusion.

Structural neuroimaging studies were included according to the following criteria: 1) used VBM to analyze whole-brain GM changes in adult (age range 18 to 60 years) MDD patients, to minimise the effect of neurodevelopment and neurodegeneration as potential confounders; 2) compared MDD patients with healthy control (HC) subjects; 3) investigated first-episode and drug naive MDD patients, who had never received antidepressant medications before MRI scanning. Functional studies were included according to the following criteria: 1) used ALFF to analyze whole-brain resting state brain activity in adult MDD patients; 2) compared MDD patients with HC; 3) investigated first-episode and drug naive MDD patients. We excluded: 1) studies from which peak coordinates could not be retrieved from the published article or after contacting the authors; 2) studies in which different thresholds were used in different regions of the brain; 3) findings based on ROIs. For studies where multiple independent patient samples were compared with HC, the appropriate coordinates were included as separate data sets. For studies using overlapping samples, the study with the most subjects was included.

Three authors (W.N.W., Y.J.Z and X.Y.H.) independently conducted the literature search. The results were compared, any inconsistencies were discussed, and a consensus decision was obtained.

### Quality assessment

We assessed the quality of the included studies using a 12-point checklist that focused on both the clinical and demographic aspects of individual study samples and on the imaging methodology. The checklist was based on previous meta-analytic studies^91, 119^, and included structural measures from MRI, modified to reflect critical variables that are important to assess VBM studies^56^ and resting state fMRI studies. This assessment included the quality of the diagnostic procedures, the demographic and clinical characterization, the prospective (or otherwise) nature of the patient and control studies, the sample size, the MRI acquisition parameters, the analysis technique and the quality of the reported results (see Supplementary Table S1). Although this checklist was not designed as an assessment tool, it provides an objective index of the rigor of individual studies. The quality scores are presented in Table 1.

### Recorded variables

For each included study we recorded: sample size, gender and mean age of subjects; illness duration, depression symptom severity and mean number of episodes; drug status; the statistical threshold of the main findings, and the method employed to correct whole-brain results for multiple comparisons. These data are presented in Tables 1 and 2.

### Standard meta-analysis of structural abnormalities

Separate voxel-based meta-analysis of regional GM abnormalities was conducted using the SDM software package^57^ (www.sdmproject.com), which implements a refinement of methods^50, 120^ which have been applied to neuroimaging studies of neurological and psychiatric disorders such as Alzheimer’s disease^34^, Attention-deficit/Hyperactivity Disorder^121^, late-life depression^56^ and MDD^58^. SDM uses the reported peak coordinates and effect sizes to recreate, based on the spatial correlation between neighbouring voxels, brain maps of the effect size of the GM differences between patient and comparison subjects, and accounts for sample size and variance as well as between-study heterogeneity. The SDM methods have been described in detail elsewhere^50, 120^, so we merely summarise the main features here. First, peak coordinates and effect sizes (derived, for example, from t values) of GM differences between MDD individuals and comparison subjects were extracted from each study. Any peaks not statistically significant at the whole brain level were excluded; thus, while different studies may employ different thresholds, we ensured that in each study the same statistical threshold was used throughout the brain. This avoids bias toward liberally threshold brain regions, which is common for ROIs. Second, a standard Montreal Neurological Institute map of the differences in GM was separately recreated for each study by means of an anisotropic Gaussian kernel, which assigns higher effect sizes to the voxels more correlated with peaks. This has been found to optimise the recreation of the effect size maps, and is robust because it does not depend on a full width at half-maximum^49^. Third, a map of the effect size variance was derived for each study from its effect size map and its sample size. Fourth, the mean map was obtained by voxel-wise calculation of the random-effects mean of the study maps, weighted by the sample size and variance of each study and the between-study heterogeneity^50^. Details of the effect size are presented in the online Supplementary Materials.

Considering possible methodological differences between the studies, we then performed subgroup meta-analyses included studies with large sample size (n > 30), studies with small sample size (n < 30), studies that utilized 1.5 T and 3.0 T MRI, studies with a correction for multiple comparisons or not, and patients with short duration (less than 6 months).

The main analysis was complemented with three analyses of robustness to ensure that only the most replicable and robust of the results were retained. First, a jackknife sensitivity analysis was performed, systematically repeating the meta-analyses excluding one study at a time: if a region remains significant in all or most of these combinations of studies, this finding is deemed highly replicable^120^. Second, a random-effects model with Q statistics was used to detect the statistical (between-studies) heterogeneity of individual clusters. Third, Egger tests were used to assess publication bias.

### Standard meta-analysis of resting state functional abnormalities

The separate main meta-analysis and the analyses of robustness of regional resting state brain activity were methodologically identical to those of regional GM.

### Multimodal Meta-Analysis

Finally, the meta-analyses of regional GM and resting state functional abnormalities were combined in order to detect those brain regions showing differences in both imaging modalities. We followed the approach described in Radua *et al*.^55^, which aims to obtain the overlap between the abnormal regions in the two modalities. In the current meta-analysis, the multimodal meta-analysis is used to detect those brain regions which display both structural and functional abnormalities. However, the exact relationship between these changes cannot be defined further.

## Electronic supplementary material

Supplementary materials
