# Supplementary material for: Conjoint and dissociated structural and functional abnormalities in first-episode drug-naive patients with major depressive disorder: a multimodal meta-analysis
Source: Sci Rep. 2017 Sep 4;7:10401. doi: 10.1038/s41598-017-08944-5 (PMC5583354; doi:10.1038/s41598-017-08944-5)
Supplement: Supplementary file 1 — Supplementary materials [file 41598_2017_8944_MOESM1_ESM.doc]

**Conjoint and dissociated structural and functional abnormalities in first-episode drug-naive patients with major depressive disorder: a multimodal meta-analysis**

Weina Wang, Ph.D.^a#^, Youjin Zhao, Ph.D.^a#^, Xinyu Hu, Ph.D.^a#^, Xiaoqi Huang, M.D., Ph.D.^a^, Weihong Kuang, M.D.^b^, Su Lui, M.D.^a^, Graham J Kemp D.Sc. (Oxf)^c^, Qiyong Gong, M.D., Ph.D.^a※^

**Author Affiliations:**

^a^Huaxi MR Research Center (HMRRC), Department of Radiology, West China Hospital of Sichuan University, Chengdu, PR China

^b^Department of Psychiatry, West China Hospital of Sichuan University, Chengdu, Sichuan, PR China.

^c^Magnetic Resonance and Image Analysis Research Centre (MARIARC) and Institute of Ageing and Chronic Disease, University of Liverpool, United Kingdom

^#^These authors contributed equally to this work.

^※^**Corresponding Author:** Prof Qiyong Gong, Huaxi MR Research Center (HMRRC), Department of Radiology, West China Hospital of Sichuan University, #37 GuoXue Xiang, Chengdu Sichuan, 610041, China; Fax: 0086-28-85423503, Tel: 0086-28-85423960. E-mail: [qiyonggong@hmrrc.org.cn](mailto:qiyonggong@hmrrc.org.cn).

**Supplementary materials**

**Table S1**

**Imaging methodology quality assessment checklist for VBM and ALFF studies*** **(Compiled from ^1-3^)**

| **Category 1: Subjects**  **Score**^§^ |
| --- |
| 1 Patients evaluated prospectively; specific diagnostic criteria applied; demographic data reported |
| 2 Healthy comparison subjects evaluated prospectively; psychiatric and medical illnesses excluded |
| 3 Important variables (e.g. age, gender, illness duration, onset time, medication status, comorbidity, severity of illness) checked, either by stratification or statistically |
| 4 Sample size per group ≥ 20 |
| **Category 2: Methods for image acquisition and analysis** |
| 5 Magnet strength ≥ 1.5T |
| 6 For VBM studies, MRI slice thickness ≤ 3 mm;  for ALFF studies, at least 5 minutes resting state acquisition |
| 7 Whole brain analysis automated with no *a priori* regional selection |
| 8 Coordinates reported in a standard space |
| 9 Imaging technique described clearly enough to be reproduced |
| 10 Measurements described clearly enough to be reproduced |
| **Category 3: Results and conclusions** |
| 11 Statistical parameters provided for significant, and important non-significant, differences |
| 12 Conclusions consistent with the results obtained; limitations discussed |
| **TOTAL**  /12 |

* Only question 6 differs between the two kinds of study.

^§^ Score: 0, criterion not met; 0.5, criterion partially met; 1, criterion fully met.

**Table S2**

Heterogeneity of VBM and ALFF changes in first-episode drug-naïve depression

| Region | MNI Coordinates | | | SDM z-score | p-value | Number of voxels |
| --- | --- | --- | --- | --- | --- | --- |
|  | X | Y | Z |  |  |  |
| Meta-analysis of VBM studies |  |  |  |  |  |  |
| Right insula | 32 | 0 | 4 | 2.352 | 0.000067115 | 300 |
| Left temporal pole | -44 | 18 | -18 | 1.123 | 0.001166344 | 87 |

*Abbreviations*: SDM, Seed-based d Mapping; MNI, Montreal Neurological Institute; VBM, voxel-based morphometry.

**Results--subgroup meta-analyses**

***VBM subgroup meta-analyses***

Considering subgroup meta-analyses of VBM studies with different sample sizes, pooled results of VBM studies are approximately the combination of the large-sample subgroup analysis and the small-sample subgroup analysis, except for right insula. For the large-sample subgroup (N>30), comprising 7 studies, the results showed increased grey matter (GM) volumes in patients in right thalamus and decreased GM volumes in right DLPFC and right SMA. For the small-sample subgroup (N<30), comprising the other 8 studies, the results showed increased GM volumes in patients in left lateral OFC, left TP, bilateral caudate nucleus and decreased GM volumes in right ITG and fusiform gyrus.

Subgroup meta-analysis of the 5 studies at 1.5 T showed increased GM volumes in the right insula and bilateral thalamus and decreased GM volumes in the right DLPFC, right SMA in MDD patients. For the 10 studies at 3.0 T, the results showed only increased GM volumes in patients in left lateral OFC and left TP.

Subgroup meta-analysis of the 13 studies with threshold correction showed increased GM volumes in patients in bilateral thalamus, right cerebellum and decreased GM volumes in the right ITG and left middle frontal gyrus in MDD patients. For the subgroup of the other 2 studies with no threshold correction, the results showed only decreased GM volumes in the right DLPFC, right SMA and right amygdala.

For subgroup meta-analysis of the 7 studies with short illness duration, the results showed partly unchanged with increased GM volumes in the left lateral OFC, bilateral thalamus and decreased GM volumes in the right ITG and middle frontal gyrus in MDD patients (Table S3).

***ALFF subgroup meta-analyses***

In subgroup meta-analysis of the 3 studies with large sample size (N>30), results remained largely unchanged with only two additional significantly increased clusters in the left posterior cingulate gyrus and right precuneus in patients. However, for the subgroup of 8 studies with small sample size (N<30), results were rather different, with increased clusters in the right putamen, right MTG, left PHG and decreased left MTG, left lingual gyrus.

Considering the different MRI B_0_ field strengths, the pooled results of VBM studies are the combination of the 1.5 T subgroup analysis and 3.0 T subgroup analysis with additional significantly increased clusters in bilateral STG at 1.5 T and a decreased cluster in left cerebellum at 3.0 T.

Subgroup meta-analysis of the 9 studies with threshold correction showed increased ALFF in patients in right putamen, right MTG, right insula and decreased ALFF in the bilateral OFC in MDD patients. For the subgroup of 2 studies with no threshold correction, the results showed only increased ALFF in the bilateral SMA and bilateral precuneus.

For subgroup meta-analyses of the 7 studies with short illness duration, the results showed increased ALFF in the right STG, right cerebellum and decreased ALFF in the left MTG, left in MDD patients precuneus (Table S4).

In addition, the main conjoint structural and functional abnormalities in left lateral orbitofrontal cortex and right supplementary motor area remained in a new multimodal meta-analysis in subgroups with large sample size (see Table S5).

**Table S3**

Regional differences in gray matter volume in subgroup meta-analyses

| Region |  | Maximum |  |  |
| --- | --- | --- | --- | --- |
|  | MNI Coordinates  x, y, z | SDM  z-score | P value  uncorrected | Number  of voxels |
| **Subgroup meta-analysis of studies with** **large sample size** |  |  |  |  |
| **MDD<HC** |  |  |  |  |
| R superior frontal gyrus, | 22, -4, 60 | -1.298 | 0.000025809 | 415 |
| dorsolateral, BA 6 |  |  |  |  |
| R supplementary motor | 16, 0, 66 | -1.254 | 0.000123858 | 195 |
| area, BA 6 |  |  |  |  |
| **MDD>HC** |  |  |  |  |
| R thalamus | 18, -26, 10 | 1.501 | 0.001104414 | 85 |
|  |  |  |  |  |
| **Subgroup meta-analysis of studies with small sample size** |  |  |  |  |
| **MDD<HC** |  |  |  |  |
| R inferior temporal gyrus, | 62,-48,-20 | -1.520 | 0.000098050 | 198 |
| BA 20 |  |  |  |  |
| R inferior temporal gyrus, | 60,-54,-18 | -1.518 | 0.000098050 | 141 |
| BA 37 |  |  |  |  |
| R fusiform gyrus, BA 20 | 42,-12,-34 | -1.588 | 0.000077426 | 129 |
| **MDD>HC** |  |  |  |  |
| R caudate nucleus | 18,24,4 | 1.237 | 0.000851512 | 59 |
| L caudate nucleus | -16,14,16 | 1.241 | 0.000784457 | 83 |
| L inferior frontal gyrus, | -44,18,-4 | 1.240 | 0.000784457 | 60 |
| orbital part, BA 47 |  |  |  |  |
| L temporal pole, superior | -52,18,-12 | 1.232 | 0.001021862 | 47 |
| temporal gyrus, BA 38 |  |  |  |  |
|  |  |  |  |  |
| **Subgroup meta-analysis of studies at 1.5 T** |  |  |  |  |
| **MDD<HC** |  |  |  |  |
| R superior frontal gyrus, | 24,-6,62 | -1.705 | 0.000314832 | 232 |
| dorsolateral, BA 6 |  |  |  |  |
| R supplementary motor | 16,-2,66 | -1.697 | 0.000356078 | 89 |
| area, BA 6 |  |  |  |  |
| **MDD>HC** |  |  |  |  |
| R insula, BA 48 | 42,0,4 | 1.040 | 0.000098050 | 479 |
| L thalamus | -6,-14,10 | 1.006 | 0.002353311 | 55 |
| R thalamus | 18,-30,6 | 1.014 | 0.001212776 | 51 |
|  |  |  |  |  |
| **Subgroup meta-analysis of studies at 3.0 T** |  |  |  |  |
| **MDD>HC** |  |  |  |  |
| L inferior frontal gyrus, | -46,20,-6 | 1.528 | 0.002074659 | 84 |
| orbital part, BA 47 |  |  |  |  |
| L temporal pole, superior | -52,16,-14 | 1.522 | 0.002146900 | 72 |
| temporal gyrus, BA 38 |  |  |  |  |
|  |  |  |  |  |
| **Subgroup meta-analysis of studies** **with threshold correction** |  |  |  |  |
| **MDD<HC** |  |  |  |  |
| R inferior temporal gyrus, | 44,-12,-34 | -1.209 | 0.000067115 | 222 |
| BA 20 |  |  |  |  |
| R inferior temporal gyrus, | 62,-50,-16 | -1.273 | ~0 | 160 |
| BA 37 |  |  |  |  |
| L middle frontal gyrus, BA 46 | -36,18,38 | -1.011 | 0.000449002 | 62 |
| **MDD>HC** |  |  |  |  |
| L thalamus | -10,-20,10 | 1.595 | 0.000588357 | 65 |
| R thalamus | 16,-28,8 | 1.661 | 0.000387073 | 76 |
| R cerebellum, hemispheric lobule VIII | 16,-68,-56 | 1.316 | 0.002864242 | 74 |
|  |  |  |  |  |
| **Subgroup meta-analysis of studies with** **no threshold correction** |  |  |  |  |
| **MDD<HC** |  |  |  |  |
| R superior frontal gyrus, | 26,0,64 | -1.659 | 0.000015497 | 362 |
| dorsolateral, BA 6 |  |  |  |  |
| R supplementary motor | 12,-2,66 | -1.637 | 0.000061929 | 155 |
| area, BA 6 |  |  |  |  |
| R amygdala, BA 34 | 20,-2,-18 | -1.066 | -1.066 | 22 |
|  |  |  |  |  |
| **Subgroup meta-analysis of studies with short illness duration** |  |  |  |  |
| **MDD<HC** |  |  |  |  |
| R middle frontal gyrus, BA 45 | 38,38,8 | -1.043 | 0.001940489 | 98 |
| R inferior temporal gyrus, | 60,-50,-16 | -1.454 | 0.000041306 | 205 |
| BA 20 |  |  |  |  |
| R inferior temporal gyrus, | 46,-10,-36 | -1.383 | 0.000123858 | 235 |
| BA 37 |  |  |  |  |
| **MDD>HC** |  |  |  |  |
| Left superior frontal gyrus, | -10,58,-18 | 1.416 | 0.003184199 | 10 |
| orbital part, BA 11 |  |  |  |  |
| L thalamus | -8,-18,4 | 1.791 | 0.000676095 | 87 |
| R thalamus | 8,-18,4 | 1.524 | 0.002188206 | 20 |

*Abbreviations:* BA, Brodmann area; GM, grey matter; HC, healthy controls; L, left; MNI, Montreal Neurological Institute Space; R, right; SDM, Seed-based d Mapping.

**Table S4**

Regional differences in brain activity in subgroup meta-analyses

| Region |  | Maximum |  |  |
| --- | --- | --- | --- | --- |
|  | MNI Coordinates  x, y, z | SDM  z-score | P value  uncorrected | Number  of voxels |
| **Subgroup meta-analysis of studies with large sample size** |  |  |  |  |
| **MDD<HC** |  |  |  |  |
| L inferior frontal gyrus, | -48,38,-6 | -1.022 | 0.000412881 | 302 |
| orbital part, BA 47 |  |  |  |  |
| R middle frontal gyrus, | 36,46,-14 | -1.025 | 0.000387073 | 277 |
| orbital part, BA 47 |  |  |  |  |
| **MDD>HC** |  |  |  |  |
| R supplementary motor | 6,-16,68 | 1.234 | 0.003870606 | 14 |
| area, BA 6 |  |  |  |  |
| R precuneus | 4,-52,36 | 1.236 | 0.003813863 | 22 |
| L posterior cingulate gyrus, BA 23 | -4,-50,30 | 1.233 | 0.003932536 | 11 |
|  |  |  |  |  |
| **Subgroup meta-analysis of studies with small sample size** |  |  |  |  |
| **MDD<HC** |  |  |  |  |
| L middle temporal gyrus, BA 21 | -66,-34,-2 | -1.253 | 0.001651466 | 391 |
| L lingual gyrus, BA 27 | -6,-48,2 | -1.894 | 0.000056744 | 84 |
| **MDD>HC** |  |  |  |  |
| R putamen, BA 48 | 36,-6,-2 | 1.362 | 0.001661777 | 113 |
| R middle temporal gyrus, BA 21 | 60,-36,0 | 1.655 | 0.000175476 | 307 |
| L parahippocampal gyrus, | -26,-12,-30 | 1.657 | 0.000175476 | 245 |
| BA 36 |  |  |  |  |
|  |  |  |  |  |
| **Subgroup meta-analysis of studies at 1.5 T** |  |  |  |  |
| **MDD<HC** |  |  |  |  |
| L inferior frontal gyrus, | -36,38,-14 | -1.350 | 0.000650287 | 265 |
| orbital part, BA 47 |  |  |  |  |
| R middle frontal gyrus, | 28,36,-16 | -1.845 | 0.000206411 | 435 |
| orbital part, BA 47 |  |  |  |  |
| **MDD>HC** |  |  |  |  |
| R superior temporal gyrus, BA 48 | 46,-22,-4 | 2.393 | 0.000005186 | 181 |
| L superior temporal gyrus, BA 48 | -50,-12,2 | 1.341 | 0.002766192 | 58 |
|  |  |  |  |  |
| **Subgroup meta-analysis of studies at 3.0 T** |  |  |  |  |
| **MDD<HC** |  |  |  |  |
| L cerebellum, hemispheric | -6,-52,0 | -2.422 | ~0 | 209 |
| lobule IV / V |  |  |  |  |
| **MDD>HC** |  |  |  |  |
| R supplementary motor | 2,-12,62 | 1.468 | 0.002441049 | 77 |
| area, BA 6 |  |  |  |  |
| L supplementary motor | -2,-6,56 | 1.465 | 0.002482355 | 56 |
| area, BA 6 |  |  |  |  |
| L parahippocampal gyrus,  BA 36 | -22,-6,-26 | 1.436 | 0.003153265 | 54 |
|  |  |  |  |  |
| **Subgroup meta-analysis of studies with threshold correction** |  |  |  |  |
| **MDD<HC** |  |  |  |  |
| L inferior frontal gyrus, | -42,40,2 | -1.179 | 0.003261626 | 103 |
| orbital part, BA 47 |  |  |  |  |
| R middle frontal gyrus, | 32,38,-16 | -1.265 | 0.002162397 | 95 |
| orbital part, BA 47 |  |  |  |  |
| **MDD>HC** |  |  |  |  |
| R putamen, BA 48 | 36,-14,-8 | 1.801 | 0.000134170 | 161 |
| R middle temporal gyrus, BA 21 | 62,-36,0 | 1.443 | 0.002152085 | 118 |
| R insula, BA 48 | 34,-8,0 | 1.743 | 0.000242531 | 347 |
|  |  |  |  |  |
| **Subgroup meta-analysis of studies with no threshold correction** |  |  |  |  |
| **MDD>HC** |  |  |  |  |
| R supplementary motor | 8,2,70 | 1.874 | 0.001450181 | 124 |
| area, BA 6 |  |  |  |  |
| L supplementary motor | 0,-16,62 | 1.877 | 0.001398563 | 98 |
| area, BA 6 |  |  |  |  |
| R precuneus | 4,-42,46 | 1.867 | 0.001615345 | 35 |
| L precuneus | -6,-44,40 | 1.871 | 0.001527607 | 23 |
|  |  |  |  |  |
| **Subgroup meta-analysis of studies with short illness duration** |  |  |  |  |
| **MDD<HC** |  |  |  |  |
| L middle temporal gyrus, BA 20 | -52,-22,-12 | -1.163 | 0.002781689 | 146 |
| **MDD>HC** |  |  |  |  |
| R middle temporal gyrus, BA 21 | 62,-40,-2 | 1.547 | 0.000371575 | 320 |
| R cerebellum, hemispheric | 26,-32,-36 | 1.460 | 0.000975370 | 54 |
| lobule IV / V |  |  |  |  |

*Abbreviations:* BA, Brodmann area; GM, grey matter; HC, healthy controls; L, left; MNI, Montreal Neurological Institute Space; R, right; SDM, Seed-based d Mapping.

**Table S5**

Regional differences in multimodal meta-analyses in subgroup with large sample size

| Region |  | Maximum |  |  |
| --- | --- | --- | --- | --- |
|  | MNI Coordinates  x, y, z | SDM  z-score | P value  uncorrected | Number  of voxels |
| **Multimodal analysis** |  |  |  |  |
| *Increased GM but* |  |  |  |  |
| *decreased brain activity* |  |  |  |  |
| L inferior frontal gyrus, | -44,24,-12 | 1.867 | ~0 | 298 |
| orbital part, BA 47 |  |  |  |  |
| *Decreased GM but* |  |  |  |  |
| *increased brain activity* |  |  |  |  |
| R supplementary motor | 14,-6,66 | 3.162 | ~0 | 587 |
| Area, BA 6 |  |  |  |  |

**Methods**

***Standard meta-analysis of structural abnormalities***

SDM uses the reported peak coordinates and effect sizes to recreate, based on the spatial correlation between neighbouring voxels, brain maps of the effect size of the GM differences between patient and comparison subjects, and accounts for sample size and variance as well as between-study heterogeneity. When a voxel can be assigned values from more than one coordinate in the same study these values are summed. An important downside of this summing of values is a bias towards studies reporting various coordinates in close proximity, as voxels can achieve rather large values. Multilevel kernel density analysis elegantly overcomes this problem by limiting the values within one study to a maximum, and SDM incorporates this feature. A novelty of our method is that both positive and negative coordinates (i.e. both increases and decreases of grey matter) are reconstructed in the same map, resulting in a signed differential map. This is an important feature that prevents a particular voxel erroneously appearing to be positive (i.e. increased volume or activation) and negative (i.e. decreased volume or activation) at the same time^4^.

Specifically, before we perform a meta-analysis in SDM software, we prepare a separate text file for each study with specified coordinates and their t statistic (a positive number represents increases of volume or brain activity; a negative number represents decreases of volume or brain activity). The Cohen’s d effect size can be calculated by the following formula^5^,


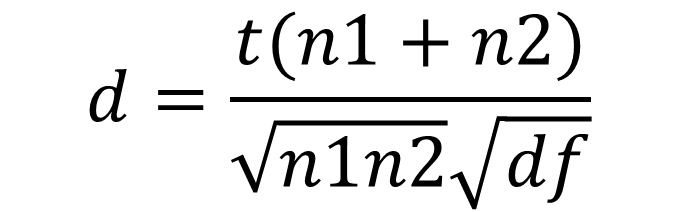


in which "df" is the degrees of freedom for the t test, and n1 and n2 are the number of cases for each group. It can be seen that if the t statistic has a particular direction, the direction of Cohen’s d value is established as the same as the t statistic.

**References**

1. Brambilla, P. *et al.* Brain anatomy and development in autism: review of structural MRI studies. *Brain Research Bulletin* **61**, 557-569, (2003).

2. Shepherd, A. M., Matheson, S. L., Laurens, K. R., Carr, V. J. & Green, M. J. Systematic meta-analysis of insula volume in schizophrenia. *Biological Psychiatry* **72**, 775-784, (2012).

3. Strakowski, S. M., DelBello, M. P., Adler, C., Cecil, D. M. & Sax, K. W. Neuroimaging in bipolar disorder. *Bipolar Disorders* **2**, 148-164, (2000).

4. Radua, J. & Mataix-Cols, D. Voxel-wise meta-analysis of grey matter changes in obsessive-compulsive disorder. *The British Journal of Psychiatry* **195**, 393-402, (2009).

5. Nakagawa, S. & Cuthill, I. C. Effect size, confidence interval and statistical significance: a practical guide for biologists. *Biological Reviews of the Cambridge Philosophical Society* **82**, 591-605, (2007).
